# A Practical Treatise on Diseases of the Skin

**Published:** 1867-02

**Authors:** 


*A Practical Treatise on Diseases of the Skin.* By J. Moore Nelligan, M. D., M. R. J. A., &c. Fifth American from the second revised and enlarged Dublin edition, by T. W. Belcher, M. A., M. D., Dublin, &c.

This work has been long approved by the profession, and is often referred to as one of the very best authorities of the skin. We think there is a want of appreciation of the importance of this subject by the profession at large. We plead guilty ourself. Every pimple has its importance in a pathological point of view. And whilst no symptoms can be set down as meaning less, they often point too, and are signifficant of the greatest trouble.

